# 6-Bromoindirubin-3’-oxime (6BIO) prevents myocardium from aging by inducing autophagy

**DOI:** 10.18632/aging.202253

**Published:** 2020-12-26

**Authors:** Donghao Guo, Lizhen Cheng, Yun Shen, Wei Li, Qinjie Li, Yuan Zhong, Ya Miao

**Affiliations:** 1 Department of Geriatrics, Shanghai Jiao Tong University Affiliated Sixth People’s Hospital, Shanghai, China; 2 Division of Cardiology, Department of Medicine and Therapeutics, Faculty of Medicine, The Chinese University of Hong Kong, Hong Kong

**Keywords:** 6-Bromoindirubin-3’-oxime, aging, oxidative stress, autophagy, rapamycin

## Abstract

6-Bromoindirubin-3’-oxime (6BIO) is a novel small molecule that exerts positive effects on several age-related alterations. However, the anti-aging effects of 6BIO on the aging heart remain unknown. Herein, we aim to investigate the effects of 6BIO on the myocardium and its underlying mechanism *in vivo* and vitro. Following 6BIO treatment, an increased p53 contents, a reduced p16 and β-gal levels, and attenuation of cardiac fibrosis were observed, suggesting 6BIO retarded aging of cardiomyocytes. As observed, 6BIO reduced p62 contents, elevated the levels of Beclin-1 and the ratio of LC3II/I, indicating the induction of autophagy, while the reduction of the accumulation of ROS indicated 6BIO alleviated oxidative stress. In addition, 6BIO treatment inhibited both GSK3β signaling and mTOR signaling. 6BIO might be a promising agent for preventing myocardium from aging.

## INTRODUCTION

Aging is a progressive deterioration of the biological structure and function of cells, tissues, or organs. Given that the prevalence of cardiovascular diseases raises significantly with increasing age [1], myocardium, according to the current mainstream view, is considered to be one of the most susceptible tissues to the age-dependent deterioration [2]. In order to prevent cardiovascular diseases and extend healthspan, current studies have now started to focus on discovering potent interventions that delay cardiac aging and investigating the possible mechanism behind it [3]. Various molecular mechanisms are involved in the age-associated alterations of cardiac structure and function. Decreased autophagy, oxidative stress accumulation, and some other alterations are commonly observed in the aging heart, which contributes to the deterioration during the aging process [4]. Interventions interacting with these molecular mechanisms and cellular processes may counteract the adverse effects of cardiac aging.

A recent report indicated that 6BIO prolonged flies’ healthy lifespan, introducing this small molecule as a novel anti-aging agent [5]. Our previous studies have revealed that a selective inhibitor of glycogen synthase kinase-3β (GSK3β), 6-Bromoindirubin-3-oxime (6BIO), is a promising agent for combating the adverse effects of hepatic aging. This small molecule also induces autophagy and attenuates oxidative stress in the liver [6]. Positive effects from 6BIO treatment have been observed in several age-associated disease models such as diabetes, neurodegenerative disease, leukemia, cancer, etc. [7–11], with several age-related molecular and cellular alterations including autophagy, inflammation, oxidative stress, cellular viability, proliferation, and apoptosis [10, 12, 13]. Recent studies have shown that 6BIO could exert therapeutic effects on some cardiovascular diseases. After myocardial infarction (MI) injury, the administration of 6BIO attenuates left ventricular remodeling [14]. Treatment with 6BIO ameliorates the cardiac micro-environment characterized by the improvement of intraventricular conduction, activation of cardiomyocytes proliferation, anti-fibrotic effects in cardiac fibroblasts, and inflammation attenuation in macrophages [15–17]. Nevertheless, treatment with the classical GSK3β inhibitor, lithium chloride, could not recapitulate the effects of 6BIO administration [17], suggesting there are some other molecular mechanisms involved in the therapeutic effects of 6BIO treatment on the heart.

Although it has been proved that 6BIO treatment ameliorates the cardiac environment in MI models and interacts with some age-associated mechanism in different organs, the effects of 6BIO on cardiac aging and its possible mechanism remains unclear. Herein, we evaluated the effects of 6BIO on the heart *in vivo* and vitro and investigated the mechanisms underlying its effects, including autophagy and oxidative stress. Our findings indicate that 6BIO treatment significantly retards cardiac aging characterized by amelioration of aging markers, suppression of myocardial fibrillation, alleviation of oxidative stress, induction of autophagy, and inhibition of mTOR as well as GSK3β. Furthermore, autophagy plays an essential role in 6BIO’s anti-cardiac aging effects.

## RESULTS

### 6BIIO and rapamycin attenuated age-associated myocardial fibrosis

**Figure 1:**
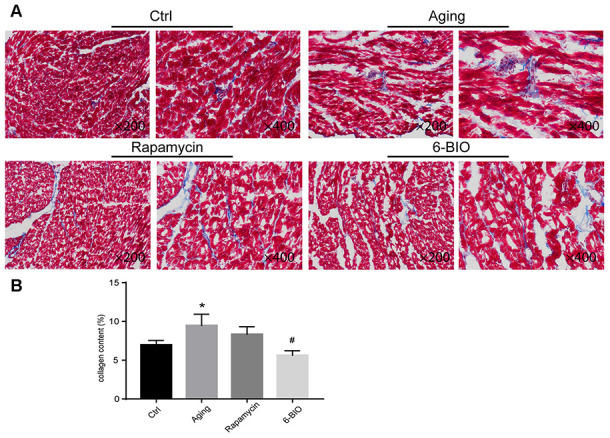
(**A**, **B**) Representative photomicrographs of Masson-stained myocardium (×200 and ×400). *P<0.05 compared with the young control group; #P<0.05 compared with the aging group.

Fibrosis, as one of the most essential indexes for evaluating cardiac remodeling, is closely related to cardiac aging [3]. Therefore, we detected the myocardial fibrosis using the Masson staining method, with blue for fibrosis. As shown in Figure 1, compared to the young control group, the aging group exhibited conspicuous fibrosis. Treatments with either 6BIO or rapamycin improved histopathological changes in aging heart tissues characterized by less fibrosis, and the effect of 6BIO was significant.

### 6BIO and rapamycin ameliorated the levels of age-related markers of aging mice

**Figure 2:**
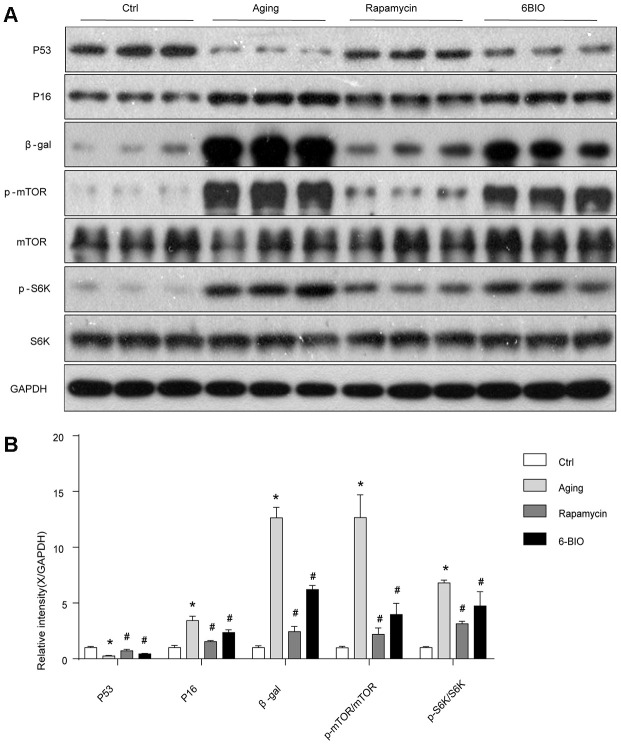
(**A**, **B**) Senescence markers and the mTOR/S6K pathway *in vivo* analyzed by Western blot. *P<0.05 compared with the young control group; #P<0.05 compared with the aging group.

To further determine the anti-aging effects of 6BIO treatment on heart tissues, age-related markers including p53, p16 and β-gal [4] were detected by Western blot. As shown in Figure 2, compared with the young control group, the cardiac tissues in the Aging group showed less p53 contents and higher p16 and β-gal levels. Rapamycin, as a potent anti-aging agent, markedly elevated p53 levels and decreased p16 and β-gal levels in aging mice. In the aging mice treated with 6BIO, a significant elevation of p53 levels and a significant reduction of p16 and β-gal contents were observed compared to the untreated aging mice. These data demonstrated the potent anti-aging effects of 6BIO on the aging heart.

### 6BIO, as well as rapamycin, negatively regulated the mTOR signaling pathway *in vivo*

The mammalian target of the rapamycin (mTOR) signaling pathway is regarded as an essential pathway associated with the aging process [18]. The levels of total mTOR and phosphorylated mTOR were detected using Western Blot. As shown in Figure 2, consistent with previous studies, the phosphorylation of mTOR was suppressed with aging, and rapamycin, as a potent inhibitor of mTOR, significantly downregulated the mTOR phosphorylation as expected. We find that 6BIO also reduced the level of phosphorylated mTOR markedly in the aging heart. In order to further assess the mTOR signaling, we detected the phosphorylation states of p70S6K, which is a downstream target of mTOR. We found that, as shown in Figure 2, the phosphorylated p70S6K increased in the aging group compared to the young control group. In the aging mice treated with either 6BIO or rapamycin, the phosphorylation of p70S6K in the aging heart significantly decreased. These results indicated that the mTOR/p70S6K signaling pathway was negatively regulated by either 6BIO or rapamycin.

### GSK3β was inhibited by 6BIO and induced by rapamycin *in vivo*

**Figure 3:**
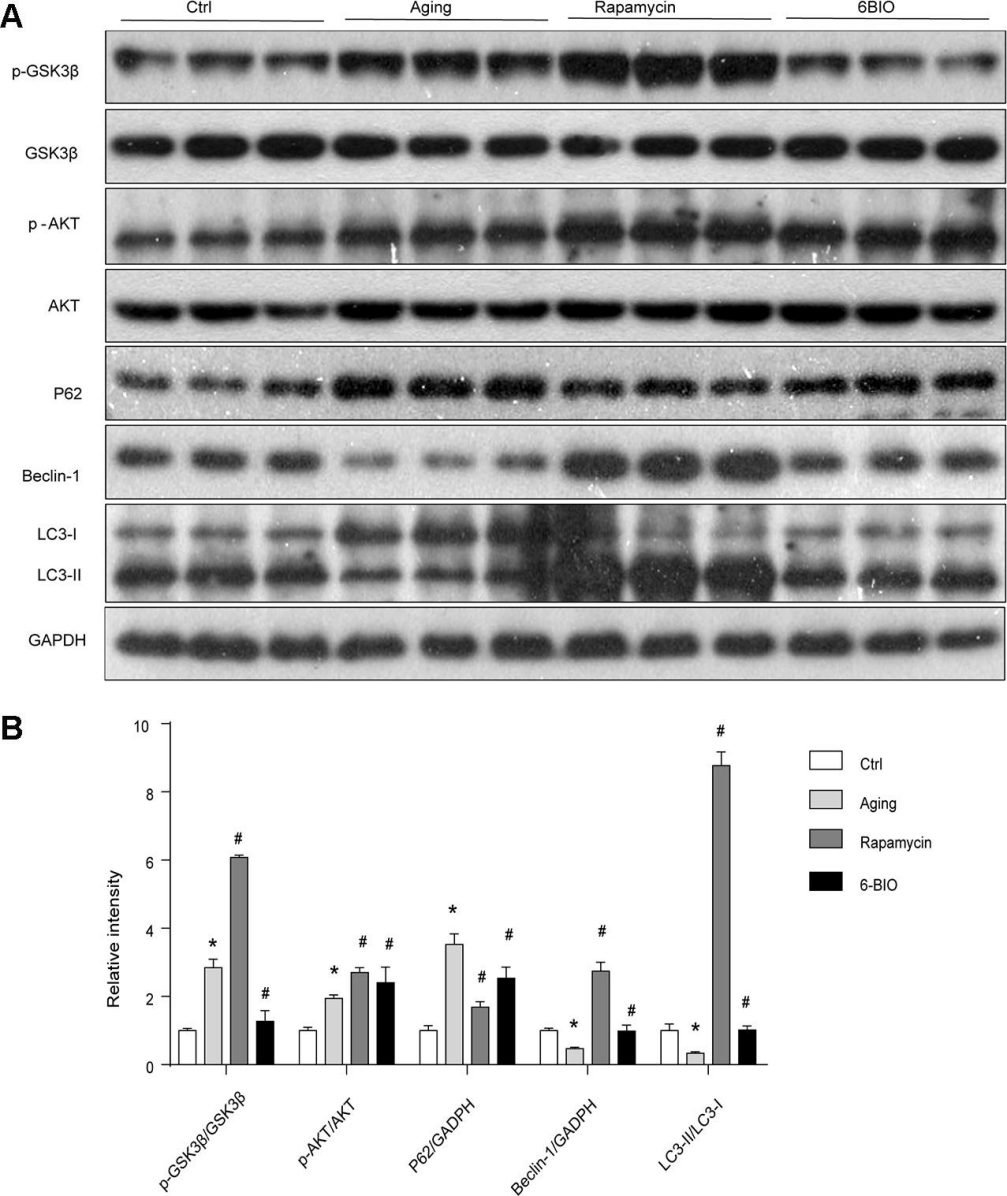
(**A**, **B**) GSK3β pathway and the activity of autophagy analyzed by Western blot. *P<0.05 compared with the young control group; #P<0.05 compared with the aging group.

As 6BIO is reported as a potent inhibitor of GSK3β [19], we used Western blot to measure the phosphorylation of GSK3β. As shown in Figure 3, The untreated aging group exhibited an evidently increased level of phosphorylated GSK3β when compared with the young group. 6BIO treatment significantly decreased the cardiac expression of p-GSK3β in aging mice. The effects of rapamycin treatment on the phosphorylation of GSK3β, however, was the opposite. We then further detected the phosphorylation of AKT, which was considered as an upstream of either GSK3β or mTOR. A significant increase of p-AKT was observed in the aging heart while after treated with 6BIO and rapamycin, the levels of p-AKT were slightly increased but the differences were not significant.

### Cardiac autophagy is enhanced by treatment with 6BIO and rapamycin

A majority of studies have revealed that autophagy plays a pivotal role in the process of aging including cardiac aging [20, 21]. Therefore, we estimated the autophagy activities by analyzing the levels of p62, Beclin-1, and LC3. As shown in Figure 3, the Western blot analysis indicated significant increased p62 contents and reduced levels of Beclin-1 and LC3II/I ratio during normal aging, implying the reduction of autophagy with aging. In response to the treatment with 6BIO and rapamycin, the significant reduction of p62 and elevation of Beclin-1 levels and LC3II/I ratio confirmed the induction of autophagy after administration.

### The oxidative stress in mice hearts was hardly alternated by 6BIO or rapamycin administration

**Figure 4:**
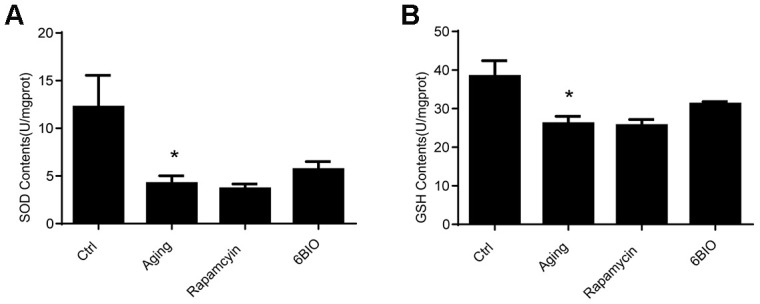
Levels of Antioxidants, (**A**) SOD and (**B**) GSH, of cardiac tissues. *P<0.05 compared with the young control group; #P<0.05 compared with the aging group.

It has been documented that oxidative stress is a vital mechanism of cardiac aging [22]. The oxidative stress damage in the heart tissues was detected in terms of the levels of SOD and GSH. As indicated in Figure 4, the activities of SOD and GSH were significantly reduced in the aging group in comparison with the young group. Nevertheless, neither rapamycin nor 6BIO could alternate oxidative stress in the aging mice hearts. Compared with the untreated aging group, the concentration of SOD and GSH was slightly elevated in the mice treated with 6BIO, but the difference was not statistically significant.

### 6BIO and rapamycin reversed H2O2-induced aging *in vitro*

**Figure 5:**
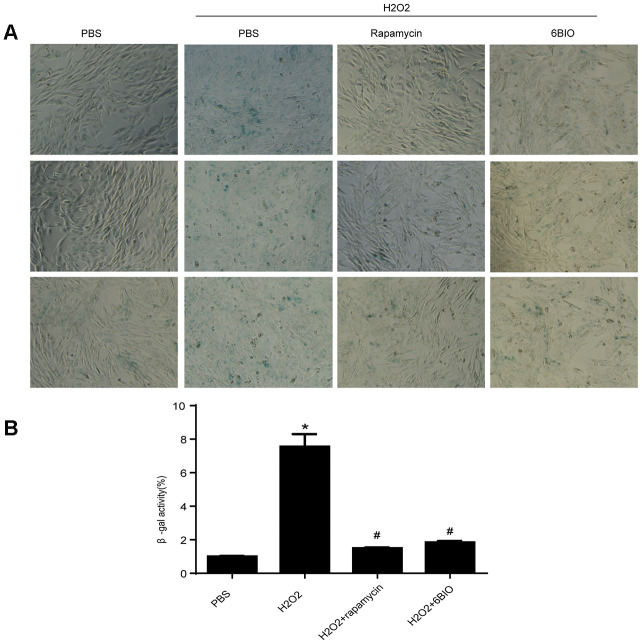
(**A**) Representative pictures of senescence-associated β -gal staining (×200) and (**B**) evaluation of senescence-associated β -gal activity assay. *P<0.05 compared with the control group treated with PBS; #P<0.05 compared with the H2O2-induced aging group.

To further investigate the effects of 6BIO on the cardiomyocytes, we conducted research on H9C2 cells. Senescence associated β-gal staining was used to determine senescence in the cell line. As shown in Figure 5, a larger amount of senescence-associated β-gal positive cells were observed in the group treated with H2O2 when compared to the control group treated with PBS, suggesting H2O2 induced cell senescence. Treatment with 6BIO and rapamycin, however, conspicuously reduced the visible blue pigment cells in the culture treated by H2O2, which implied the reverse of myocardial senescence.

**Figure 6:**
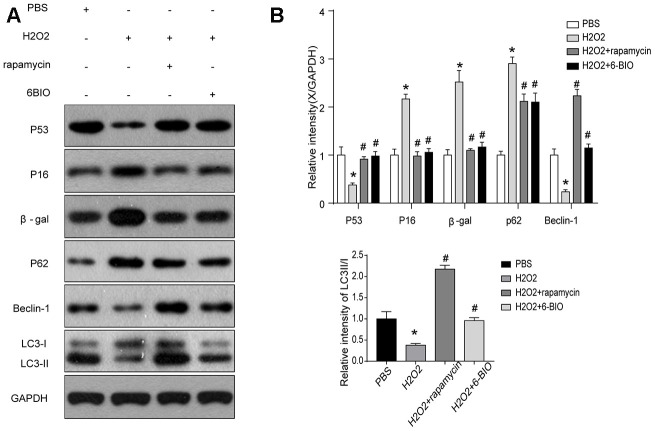
(**A**, **B**) Senescence markers and the activity of autophagy *in vitro* analyzed by Western blot.

We then analyzed the concentration of some senescence markers using Western blot and found that H2O2 evidently reduced P53 contents and increase the concentration of P16 and β-gal, and treatments with 6BIO and rapamycin reversed the effects of H2O2 on the expression levels of these proteins. These results suggested that H2O2-induced aging in myocardial cells could be reversed by 6BIO as well as rapamycin (Figure 6).

### Autophagy was induced by 6BIO and rapamycin *in vitro*

Next, we used Western blot to evaluate autophagic alterations by detecting autophagy specific markers. As shown in Figure 6, H9C2 cells treated by H2O2 exhibited a marked increase in the expression levels of p62 and a notable reduction in the concentration of Beclin-1 and the ratio of LC3II/I, suggesting the suppression of autophagy. The p62 contents were elevated and the levels of Beclin-1 and the ratio of LC3II/I were reduced in the group treated with 6BIO and rapamycin compared to the group treated with H2O2 only, indicating the induction of autophagy *in vitro*, which was consistent with the results of previous animal experiments.

### The accumulation of ROS was suppressed following 6BIO treatment and rapamycin treatment

**Figure 7:**
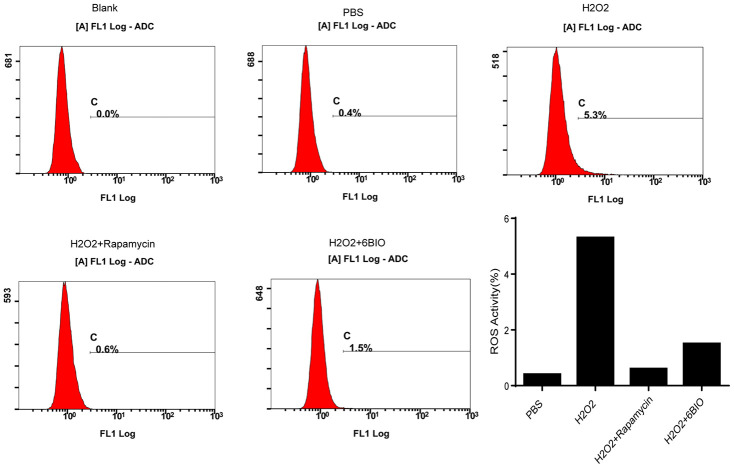
**The measurement of ROS generation in H9C2 cells.**

It has been established that ROS contributed to oxidative stress and made influences on cell states [23]. As indicated in Figure 7, compared with the control group, an accumulation of ROS has been observed in the cardiomyocytes treated with H2O2, while 6BIO and rapamycin treatment restrained the accumulation of ROS induced by H2O2.

### 6BIO inhibited both mTOR and GSK3β in cardiomyocytes

**Figure 8:**
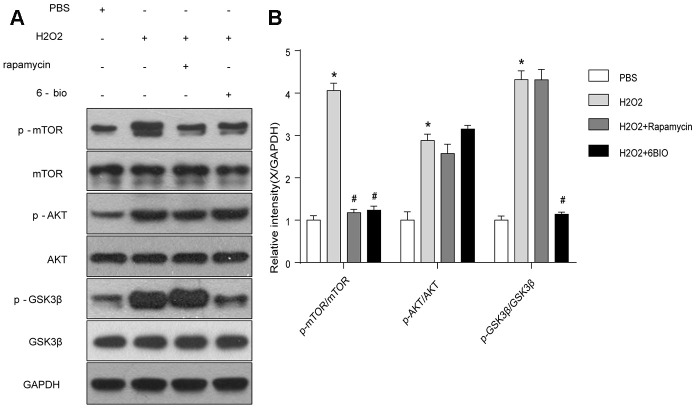
**The mTOR and GSK3β signaling analyzed by Western blot.** *P<0.05 compared with the control group treated with PBS; #P<0.05 compared with the H2O2-induced aging group.

To further investigate the potential pathway involving in the anti-aging effects of 6BIO, we analyzed the contents of total and phosphorylated mTOR, AKT, and GSK3β. As indicated in Figure 8, the phosphorylation of mTOR, AKT, and GSK3β was significantly enhanced by H2O2 treatment. 6BIO, as well as rapamycin, suppressed the phosphorylation of mTOR induced by H2O2. There was no significant difference observed in the expression levels of p-AKT following 6BIO and rapamycin treatment. 6BIO evidently inhibited the phosphorylation of GSK3β, while rapamycin exerted no significant effects on that.

### 6BIO exerted its anti-aging effects on cardiomyocytes by inducing autophagy

To further demonstrate the role of autophagy in 6BIO’s anti-aging effects, we applied chloroquine (CQ) to inhibit autophagy by blocking the fusion of autophagosomes and lysosomes and inspected the changes. In the meantime, we further detected the autophagic flux based on the RFP and GFP intensity.

**Figure 9:**
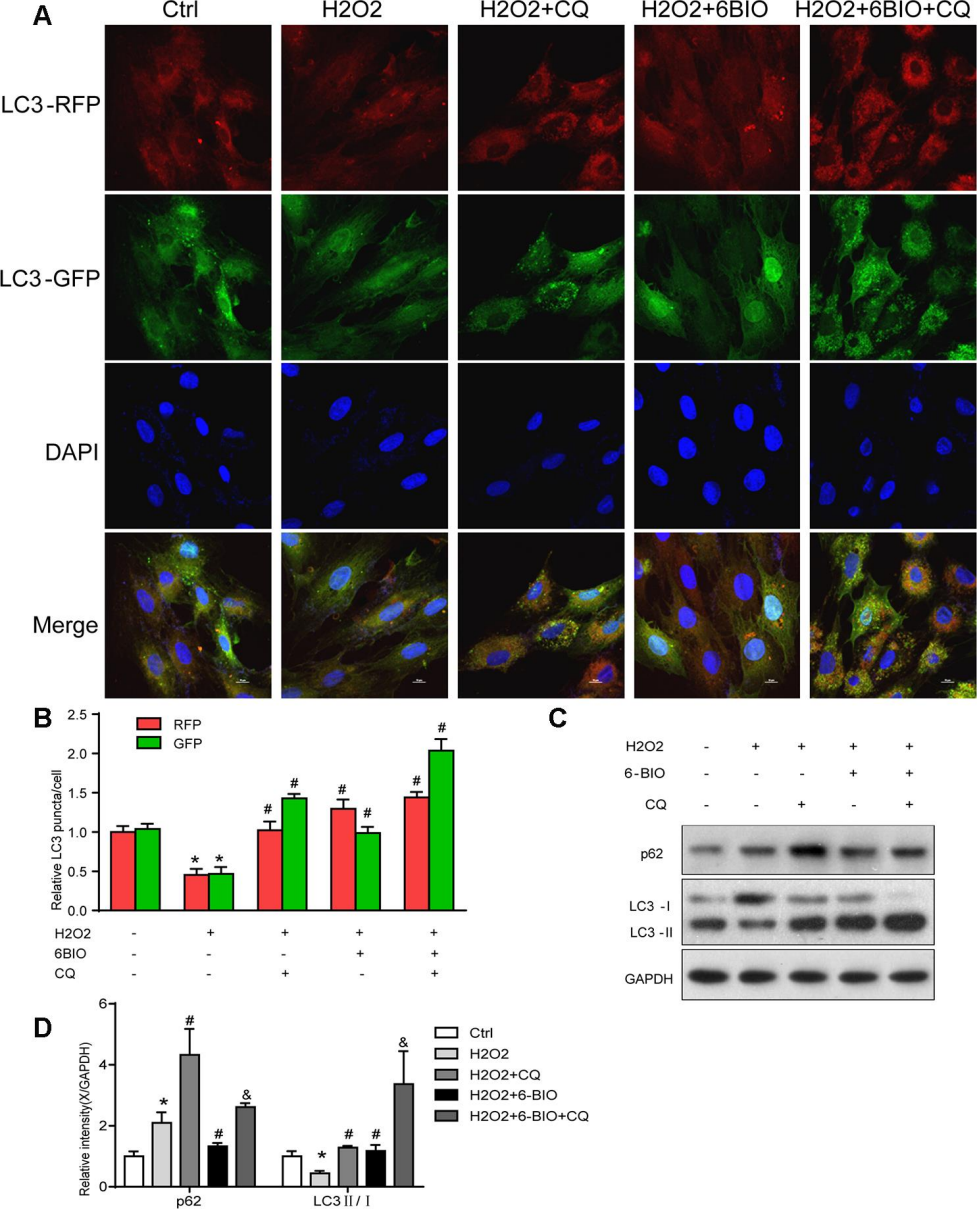
**The autophagy flux analysis.** (**A**) Represent images were captured under fluorescence microscope with 1000× magnification. Autophagosomes were labelled by both green and red punctate dots and autolysosomes were labelled by red puncta only. (**B**) Quantification of relative LC3 puncta was plotted. (**C**, **D**) The autophagy-related proteins were assessed using Western blot. *P<0.05 compared with the control group treated with PBS; #P<0.05 compared with the H2O2-induced aging group; &P<0.05 compared with the H2O2+CQ group.

**Figure 10:**
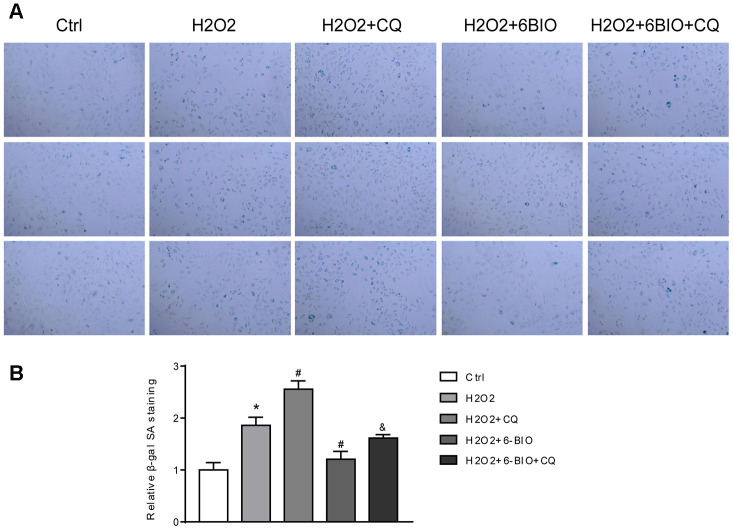
(**A**) Representative pictures of senescence-associated β -gal staining (×200) and (**B**) evaluation of senescence-associated β -gal activity assay. *P<0.05 compared with the control group treated with PBS; #P<0.05 compared with the H2O2-induced aging group; &P<0.05 compared with the H2O2+6BIO group.

RFP is acid insensitive, while GFP is acid-sensitive which is quenched in the acidic autolysosome. Thus, the progression from the autophagosome to autolysosome can be visualized under a fluorescence microscope. As shown in Figure 9A, 9B, compared to the control group, a significant reduction of the total amount of autophagosome and autolysosome has been observed, indicating the inhibition of autophagy at the early stage. The increased ratio of GFP to RFP in the group co-treated with CQ confirmed that the formation of autolysosome was blocked by CQ. The group co-treated with 6BIO showed a marked increase in the amount of RFP and GFP as well as the ratio of GFP to RFP, suggesting the elevated autophagic flux. Compared to H2O2 + CQ group, the H2O2 + 6BIO + CQ group exhibited more total amount of autophagic vacuoles, further verifying that 6BIO augmented autophagic flux. Also, we used Western blot to confirm autophagic alterations. As shown in Figure 9C, 9D, consistent with the fluorescence results, H2O2 treatment downregulated the autophagy, while 6BIO upregulated autophagy. CQ blocked autophagy at the late stage causing the accumulation of LC3-II. Compared to H2O2 + CQ treatment group, the group co-treated with H2O2, 6BIO, and CQ showed markedly lower P62 contents and a higher ratio of LC3-II/I, confirming that 6BIO augmented autophagy flux significantly and CQ blocked autophagy at the late stage. We then performed senescence-associated β-gal staining to detect senescence in the cell line. As shown in Figure 10A, 10B, H2O2 treatment induced more expression of senescence-associated ß-galactosidase, while 6BIO co-treatment reduced the expression of senescence-associated ß-galactosidase. CQ, which blocked autophagy, eliminated the effects of 6BIO. These results indicated that 6BIO retarded aging by inducing autophagy in cardiomyocytes.

## DISCUSSION

Accumulating data has illustrated that cardiovascular diseases have become the most common reason causing death in old age people [24]. Cardiac aging characterized by age-dependent structural and functional cardiac alterations, to some degree, contributes to the development of cardiovascular diseases [25]. However, the principal changes in the aging heart during aging and its mechanisms have not been well identified [26]. In this study, we investigated changes during normal aging in mice models and changes resulting from H2O2-induced aging in myocardial cells, including histopathological changes, senescence markers contents alterations, and molecular biological changes. Our results reveal that normal aging causes significant alterations of cell senescence markers and aggravates myocardial fibrosis. The molecular biological status of the myocardium is sensitive to increasing age, which explains the prevalence of cardiovascular diseases in elders, to some degree. These changes of senescence-associated markers also indicated that the aging models have been successfully established and these markers can be used for estimating the anti-aging effects of the drug candidates. In addition, the observed changes in the expression levels of some proteins imply some possible mechanisms related to cardiac aging. These findings indicate that autophagy, oxidative stress, mTOR pathway, and GSK3β pathway all contribute to the deterioration of the heart condition during aging.

With a better understanding of the aging mechanism, anti-aging pharmacology enables people to prevent alterations in the cardiovascular system from aging rather than deal with all the aging-associated complications one by one [27]. Recent studies have demonstrated that the natural compound derivative, 6BIO, exerted protective effects on the heart after myocardial infarction [14, 17]. Nonetheless, little research has focused on the anti-aging effects of 6BIO on the aging heart. Over the past decades, more and more hallmarks of aging have been revealed. As a result, monitoring aging has become more practical. In this study, we examined several molecular senescence markers as well as some age-dependent changes to identify the effects of our candidate drug. Amazingly, most of the biomarkers we examined were significantly ameliorated following 6BIO treatment. Our research found that aging led to cardiac fibrosis, meanwhile, 6BIO reduced fibrosis in myocardium resulting from aging. The results of cell senescence markers including p53, p16, and β-gal were also altered in a positive manner by 6BIO treatment. The elevated levels of p53 and the attenuation of p16 and β-gal contents in the group treated by 6BIO *in vivo* and vitro confirmed that the degree of cell senescence was markedly improved. To our best knowledge, our studies for the first time recognized 6BIO as a potent agent for preventing cardiac aging.

It has been well established that autophagy and oxidative stress both play pivotal roles in cardiac aging [28, 29]. In addition, it has been recently reported that 6BIO attenuates oxidative stress in skin fibroblasts and livers [6, 30] and it is a novel inducer of autophagy in neurons and livers [6, 13]. However, the effects of 6BIO on autophagy and oxidative stress in myocardium remain uncertain. Herein, our results indicated that 6BIO induced cardiac autophagy in aging mice and in H2O2-induced aging cardiomyocytes, which possibly contribute to the anti-aging effects of 6BIO. As observed, the ROS-induced oxidative damage was alleviated significantly by treatment with 6BIO and rapamycin in H9C2 cells. By contrast, the differences in the levels of an antioxidant enzyme, SOD and a vital catalase, GSH following 6BIO treatment and rapamycin treatment in mice were not significant, which must be carefully inspected from different perspectives given that the effects of 6BIO range from indirect regulations like in the expression levels to a direct physical interaction [29]. Nevertheless, the mechanism underlying 6BIO’s anti-oxidant effects on cardiomyocytes remains uncertain. ROS are formed as a natural byproduct strongly influenced by stress factors. An imbalance between the production of ROS and the anti-oxidant system might bring cellular damages, which is known as oxidative stress. The anti-oxidant machinery consists of the enzymatic anti-oxidants, like SOD, and non-enzymatic anti-oxidants, such as GSH. As our study showed dissimilar results of ROS and anti-oxidant protein, implying that 6BIO might not reduce ROS via interacting with the anti-oxidant enzymes like SOD or GSH, there is a possibility that 6BIO exerts its anti-oxidative effects by interfering the non-antioxidant metabolites. For instance, the production of ROS might be hindered during the metabolism of oxygen in mitochondria, plasma membranes, peroxisome, or endoplasmic reticulum, where NADPH oxidase, xanthine oxidase (XOD), and etc. might involve in this process [31]. However, the exact mechanism of 6BIO’s anti-oxidative effects requires better explored.

According to our results, the sole inhibition of autophagy flux increases the induction of senescence by H2O2. This result alone does not allow us to confirm the autophagy participation in the protection. However, increasing evidence suggests that autophagy is an essential anti-senescence mechanism of cardiac aging in either vivo or vitro experiments [32, 33]. We suppose that this result might come from the fact that the cellular senescence model induced by H2O2, which is one of the most widely used vitro models for aging research, might be regarded as a senescence model mainly induced by oxidative stress [34]. Although in our results, H2O2 treatment for 24 hours also suppressed autophagy as well, autophagy might only be a branch of the complex molecular network with crosstalk in this H202-induced senescence model. Previous studies showed that in the senescent cell model induced by short-term (3 hours) H2O2 treatment, autophagy was induced as a cytoprotective mechanism [35]. Hence, when the perturbation of autophagy induced by CQ further eliminated the cytoprotective effect of autophagy, the induction of senescence by H2O2 was enhanced. We assume that autophagy might play a role in preventing aging in the context of H2O2 treatment, but the interaction between complex molecular networks needs further study. It has been documented that 6BIO is a potent inhibitor of GSK3β and rapamycin is a potent mTOR inhibitor [5, 36]. Besides, mTOR pathway and GSK3β pathway are reported to play vital roles in autophagy and oxidative stress, and the attenuation of both signaling pathways delay the aging process [5, 37]. It is logical to investigate the mTOR pathway and GSK3β pathway. Our results showed that rapamycin impeded mTOR signaling, which was consistent with previous studies but failed to retrain GSK3β signaling. 6BIO, by contrast, inhibited both GSK3β signaling and mTOR signaling, suggesting that 6BIO might exert its cardiac anti-aging effects via interaction with either GSK3β or mTOR.

In conclusion, this study indicates that 6BIO exerted cardiac anti-aging effects *in vivo* and vitro as well as rapamycin, one of the most promising anti-aging agents [38], characterized by the amelioration of age-related changes including the attenuation of fibrosis, the amelioration of cell senescence markers, the induction of autophagy, and the alleviation of oxidative stress. Although the underlying mechanism by which 6BIO exerted its cardiac anti-aging effects still requires further investigation, our studies suggest that 6BIO might induce autophagy and attenuate oxidative stress to retard the aging process in the aging heart through inhibition of both GSK3β and mTOR signaling.

## MATERIALS AND METHODS

### Animals and treatment

We obtained male young (2-month-old) and aging (18-month-old) C57BL/6J mice from the Experimental Animal Center of the Chinese People’s Liberation Army Fourth Military Medical University. All mice were maintained in a specific pathogen-free (SPF) environment with light and temperature control and had ad libitum access to food and water. We divided the experimental mice into the following four groups, with eight mice per group: (1) Young control-young male mice injected intraperitoneally with 0.9% NaCl(10 ml/kg) every day, (2) Aging control-aging male mice injected intraperitoneally with 0.9% NaCl(10 ml/kg) every day, (3) Rapamycin treatment-aging male mice with injection by rapamycin (4 mg/kg) in 10ml/kg of saline every day intraperitoneally [39]. (4) 6BIO treatment-aging male mice received treatment with 6BIO (10 mg/kg) in 10ml/kg of saline every day intraperitoneally [13], For the reason that rapamycin is reported to reverses age-related cardiac dysfunction, we use rapamycin treatment as the positive control by promoting mTOR-regulated autophagy [40]. After a 2-week administration, the mice were sacrificed. The heart samples were cut into two parts, one of which was stored at -80° C for subsequent analysis, the other one of which was fixed in 4% paraformaldehyde for 48 hours and embedded in paraffin. All animal studies were approved by the Animal Experimentation Ethics Committee of Shanghai Jiao Tong University Affiliated Sixth People’s Hospital following the guidelines of the Institutional Animal Care and Use Committee (IACUC) of Shanghai Jiao Tong University.

### Determination of antioxidant enzyme activities and lipid peroxidation

Heart tissues were minced and immersed in nine volumes of cold phosphate-buffered saline (PBS) to homogenize. Homogenates were centrifuged at 3000 rpm for 15 minutes at 4° C to obtain the supernatants for the estimation of oxidative stress markers. Superoxide dismutase (SOD) activities and glutathione (GSH) activities were measured according to the instruction of commercial assay kits purchased from the Nanjing Jiancheng Institute of Biotechnology (Nanjing, China).

### Masson staining

Heart tissues were fixed in 4% paraformaldehyde for 48 hours and then were dehydrated in gradient alcohol, vitrified with dimethyl benzene, and embedded in paraffin. Next, the heart samples were sliced into 5μm-thick sections. Masson’s trichrome staining was performed according to standard protocol, and all the sections were observed using a microscope at magnifications of ×200 and ×400.

### Cell culture and treatment

H9C2, a ventricular myoblast cell line derived from embryo, was obtained from the American Type Culture Collection (Manassas, VA, USA) and cultured in Dulbecco’s modified Eagle’s medium (Invitrogen, Carlsbad, CA, USA) supplemented with 10 % fetal bovine, 2 mM l-glutamine, 0.1 mM nonessential amino acids, 100 units/ml of penicillin, and 100 μg/ml of streptomycin at 37° C in a humidified 5% carbon dioxide (CO2) incubator. After 24 hours incubation, cells had become attached to the plate and were treated with the desired experiment conditions: (1) Control group-treated with PBS, (2) Aging group-treated with H2O2 (150μM) for 24 hours, (3) Rapamycin treatment group-treated with H2O2 (150μM) and rapamycin (100nM) for 24 hours, (4) 6BIO treatment group-treated with H2O2 (150μM) and 6BIO (10μM) for 24 hours, (5) H2O2 + CQ group-treated with H2O2 (150μM) and CQ (50 μM) for 24 hours, (6) H2O2 + 6BIO +CQ group-treated with H2O2 (150μM), CQ (50 μM), and 6BIO (10μM) for 24 hours. After treatment, the cells were harvested for further analysis.

### Western blotting

The heart tissues and the H9C2 cells were homogenized and lysed with a lysis buffer (RIPA with protease and phosphatase inhibitor). Total protein concentrations were determined using bicinchoninic acid protein assay kits (Beyotime Biotechnology, China). Protein samples were separated by sodium dodecyl sulfate-polyacrylamide gel electrophoresis (SDS-PAGE) and transferred onto polyvinylidene difluoride membranes (PVDF). After blocked with 5% skim milk for 1 hour at room temperature and incubated overnight with the appropriate antibodies at 4 ° C, the membranes were then incubated with horseradish peroxidase-labeled secondary antibody (1:5000; Bio-Rad, USA) for 1 hour at room temperature. The primary antibodies included mTOR rabbit mAb (1:1000; Cell Signaling Technology, USA), phospho-mTOR rabbit mAb (1:1000; Cell Signaling Technology, USA), p70 S6 kinase rabbit mAb (1:1000; Cell Signaling Technology, USA), phosphor-p70 S6 kinase rabbit mAb (1:1000; Cell Signaling Technology, USA), GSK-3β rabbit mAb (1:1000; Cell Signaling Technology, USA), phospho-GSK3β(Ser9) rabbit mAb (1:1000; Cell Signaling Technology, USA), AKT rabbit mAb (1:1000; Cell Signaling Technology, USA), phospho-AKT rabbit mAb (1:1000; Cell Signaling Technology, USA), P53 rabbit mAb (1:1000; Cell Signaling Technology, USA), P16 rabbit mAb (1:1000; Cell Signaling Technology, USA), β-gal rabbit mAb (1:1000; Cell Signaling Technology, USA), LC3I/II rabbit mAb (1:1000; Cell Signaling Technology, USA), P62 rabbit mAb (1:1000; Cell Signaling Technology, USA), beclin-1 rabbit mAb (1:1000; Cell Signaling Technology, USA), and GAPDH rabbit mAb (1:1000; Cell Signaling Technology, USA). Lastly, the antigen-antibody bands were visualized by enhanced chemiluminescence (ECL) method using an electrochemiluminescent solution (Millipore, USA) and the ImageJ (NIH, USA) analysis system.

### Measurement of reactive oxygen species (ROS)

After washed with PBS, the collected cardiomyocytes were incubated with 5 μM 5-(and-6)-chloromethyl-20,70-dichlorodihydrofluorescein diacetate acetyl ester (DCFDA) at 37° C for 30 minutes. Levels of intracellular ROS were estimated as the fluorescence of DCF by flow cytometry with an excitation wavelength of 480 nm and an emission wavelength of 535 nm [23]. Tracings were obtained via displaying the log fluorescence of the samples generated against the background staining of cells.

### Senescence β-galactosidase (β-gal) staining

The levels of senescence β-gal were determined using a senescence β-gal staining kit (Cell Signaling Technology, USA) following the manufacturer’s protocol. After treated with respective intervention, cells were incubated with a β-gal staining solution at 37° C overnight. Senescent cardiomyocytes were identified as the blue-stained cells under an Olympus IX51 inverted microscope.

### Tandem RFP-GFP-LC3 fluorescence microscopy

For analysis of autophagy flux, a tandem fluorescent-tagged RFP-GFP-LC3 adenovirus (Shanghai Asia-Vector Biotechnology, China) was used to infect H9C2 cells. After incubating with RFP-GFP-LC3 adenovirus for 24 hours, the cells were subjected to corresponding treatment for an additional 24 hours. Next, cells were fixed with 4% paraformaldehyde, followed by DAPI staining visualizing nuclei. Representative images were captured under the fluorescence microscope with 1000× magnification. Autophagosomes were labeled by both green and red punctate dots and autolysosomes were labeled by red puncta only. Image analysis was performed using ImageJ (NIH, USA) software.

### Statistical analysis

Statistical analysis was performed using the GraphPad Prism 7.0 software. Results were presented as mean ± standard deviation (SD). One-way ANOVA analysis and Tukey post hoc analysis were used to assess statistical significance; p values less than 0.05 were considered statistically significant.
